# Awareness and Malaria Prevention Practices in a Rural Community in the Ho Municipality, Ghana

**DOI:** 10.1155/2019/9365823

**Published:** 2019-05-21

**Authors:** Kennedy Diema Konlan, Hubert Amu, Kennedy Dodam Konlan, Milipaak Japiong

**Affiliations:** ^1^Department of Public Health Nursing, School of Nursing and Midwifery, University of Health and Allied Sciences, Ho, Ghana; ^2^Department of Population and Behavioural Sciences, School of Public Health, University of Health and Allied Sciences, Hohoe, Ghana; ^3^Department of Nursing, West End University College, Ghana; ^4^Department of Nursing, School of Nursing and Midwifery, University of Health and Allied Sciences, Ho, Ghana

## Abstract

**Background:**

Malaria is no doubt a burden on both the financial and human resources of Ghana. In this study, we examined the awareness of malaria prevention practices among indigenes of Godokpe, a rural community in the Ho Municipality of Ghana.

**Methods:**

This descriptive cross-sectional survey that used a self-developed questionnaire recruited 246 residents of Godokpe who were aged 18 years and above using a systematic sampling technique. The Statistical Package for Social Sciences (SPSS) was used to analyze data into descriptive and analytical statistics. The descriptive statistics comprised frequency, percentage, and means. Also, analytical statistics of cross tabulation was conducted considering a p<0.05 at a 95% Confidence Interval to be statistically significant.

**Findings:**

About 54% and 20% of the respondents, respectively, had satisfactory and good levels of knowledge on malaria prevention. The methods used in malaria prevention included mosquito coils (72%), cleaning and prevention of water stagnation (62%), mosquito spray (54%), and mosquito net (59%). Also, malaria treatment methods mostly used were quinine (70%) and chloroquine (50.4%). The major sources of information on malaria were television (74%), health professionals (66%), schools (62%), family/friends (60%), and the Internet (51%).

**Conclusion:**

School children showed good understanding of malaria and its vectors. There is, therefore, the need to increase the empowerment of teachers with appropriate health information including malaria so that they can continue to deliver malaria information to the pupils.

## 1. Introduction

Malaria continues to be a serious public health problem in sub-Saharan Africa [1] and affects the health and wealth of nations and individuals alike [2]. Children aged less than five years and pregnant women are the people most vulnerable to dying of malaria or suffering serious consequences of the disease, especially in regions where transmission is intense [3, 4]. Children are most vulnerable because they have not yet acquired immunity to the disease, while maternal susceptibility to malaria infection during pregnancy may be related to the physiological immunosuppression that occurs during gestation. In children under five, the adverse effects include convulsions, anaemia, coma, and death. In Ghana, preventing malaria is of prime importance in reducing the rates of morbidity and mortality [5]. Malaria is actually responsible for about 31% of consultations, 44% of hospitalizations, and 18% of deaths occurring in health facilities in the country [5]. In children less than 5 years, 41% of deaths are due to malaria [5]. If malaria is appropriately prevented, the individual, family, and the state will save lots of resources that will improve the standards of living of the general population [5].

Malaria is unique among diseases because its roots lie so deep within human communities [6]. Beliefs and practices related to malaria are often associated with culture and can influence the effectiveness of control practices [7]. Similarly, Kimbi et al. [5] note that the practice of malaria preventive is related to the level of knowledge and belief of people. The understanding of the possible causes, modes of transmission, and decision about adoption of preventive and control measures vary from community to community and among individual households. Nkuo-Akenji et al. [8] reported that an adequate knowledge of mothers of under-fives about malaria has a great correlation with reduced morbidity and mortality among children less than five years. However, a lot of misconceptions concerning malaria still exist. Thus, local knowledge and practices related to malaria are important for the implementation of culturally appropriate, sustainable, and effective interventions (Vijayakumar, 2009). Malaria cannot be controlled by the health sector alone; therefore, multiple strategies must be pursued with other health related sectors. In view of this, Ghana implemented interventions to help in the control of the deadly disease. Some of the interventions applied included residual insecticide application against adult mosquitoes, mass chemoprophylaxis with Pyrimethamine medicated salt, and improvement of drainage system. But malaria continued to be the leading cause of morbidity (illness) in the country [9]. Overall, the Ghana Roll Back Malaria programme emphasizes the strengthening of health services through multi- and intersectoral partnerships and making treatment and prevention strategies more widely available [10].

Government has taken several initiatives in the prevention of malaria and these include the free distribution of Long Lasting Insecticide Treated Nets (LLITNs) to pregnant women and children five (5) years and below, the subsidizing of the cost of LLITNs to the rest of the population, the subsidizing of the cost of artemisinin-based combination therapy used as first line treatment for uncomplicated malaria cases, and training in the community of local health assistance capable of managing uncomplicated malaria cases and providing adequate advices [10].

Despite the measures put in place by the government and its partners, malaria still remains a public health concern, a major cause of mortality and morbidity especially in pregnant women, nursing mothers, and children under five years in Ghana [11]. In the Volta region of Ghana, malaria topped the morbidity indicators recording 617,191 cases representing 40.96% of the total Outpatient Department (OPD) attendance (GSS, 2010). Ho municipality recorded 70,567 cases in 2014 (Ho Municipal Health Directorate, 2016). From Ho Polyclinic annual review (2016), 70% of the malaria cases reported come from Godokpe community. Significant numbers of such cases were recorded in both pregnant women and nursing mothers. With the above figures showing devastating rate of malaria infection in Ho municipality, this study seeks to investigate the awareness of malaria prevention among people living in Godokpe community.

## 2. Material and Methods

### 2.1. Setting

The study was conducted among residents of Godokpe community, Ho, in the Volta region of Ghana. Ho is one of the 25 districts/municipalities in the Volta region of Ghana (Spear & Lock, 2003). The population of the municipality is 96,213 (GSS, 2010). The population density of the municipality in 2010 was 281.2 persons per square kilometer. Ho municipality has 46.7% of the population in urban locality and the remaining 53.3% are in the rural locality (GSS, 2010). Ho has an average temperature and humidity of 27 degrees Celsius and 82%, respectively (Deutscher Wetterdienst). Godokpe has only one health facility; thus, the Ho polyclinic serves the healthcare needs of the inhabitants. Godokpe lies on latitude 6.6023 degrees in the North and 0.4844 degrees in the east. The estimated population growth rate of the Ho municipality which includes Godokpe is 17.8% (GSS, 2010). There is a 2.3km feeder road from the township to Godokpe junction and there are minor roads linking other areas of the community (GSS, 2010).

### 2.2. Design and Population

The study employed a descriptive cross-sectional design as data was collected from study participants once and no follow-up was carried out. The study targeted all persons living in Godokpe community. The actual population was 788 inhabitants (GSS 2010). Out of this number, 376, 233, and 179 were females, males, and children under 18 years, respectively (GSS, 2010). This study involved all people who were aged eighteen years and above living in Godokpe community at the time of study.

### 2.3. Sampling and Data Collection

With this, items are selected at random from the population and used to test the hypotheses about the population.

Sample size was calculated using Cochran formula for sample size calculation.

n_o_ = *z*^2^*pq*/*e*^2^; substituting these figures into the Cochran equation,

n_o_ = (1.96^2^ × 0.8 × 0.2)/0.05^2^ = (3.8416 × 0.8 × 0.2)/0.0025 = 0.614656/0.0025 = 245.8624≈246

∴ n= 246.

Hence 246 respondents were recruited to respond to a questionnaire.

A systematic sampling strategy was employed to select subjects for the study. The total adult population of the community was 609 adults that were qualified to participate in the study. This produced sample factor of 2.5. Recruiting participants for the study every 2nd or 3rd person (depending on which was convenient) within the community was recruited for the study. We preferred this sampling procedure because it was fast and inexpensive and the study subjects were readily available. A questionnaire was administered to selected community members in Godokpe.

A self-developed questionnaire was used to collect data from the respondents. The questionnaire was subdivided into five parts with Part 1 focusing on the sociodemographic data of respondents and Part 2 pertaining to the awareness on malaria spread, symptoms, and treatment. Part 3 contained questions which elicited information on knowledge of preventive measures whilst Part 4 involved questions intended to identify strategies/materials used in malaria prevention. Part 5 also had questions on sources of information on various aspects of malaria fever. The questionnaire was interpreted to respondents who could not read and write by trained research assistants.

In an attempt to ensure that the research questions were not ambiguous, the questionnaire was pretested on forty people living in Ho-Bankoe and the data collected was subjected to a reliability test on SPSS version 22. This was done to ascertain respondents' general reaction and, particularly, interest in answering the questionnaire. The questionnaire was modified until it produced a* Cronbach Alpha coefficient* of 0.701. It can, therefore, be concluded that the instrument had a high reliability in measuring needed data for the study. Responses from the people showed that the questionnaire was clear and could be understood by others.

### 2.4. Data Handling

Collected questionnaires were checked for completeness and appropriateness of responses before they were then coded. Completed questionnaires were entered into the Statistical Package for Social Sciences (SPSS) version 22. The data was cleaned to ensure the quality of the entered data before the analysis.

### 2.5. Data Analysis

Numerical data was analyzed using descriptive statistics. Cross-tabulation analysis was performed to determine association between categorical variables, and* P* value of less than 0.05 significance level was considered statistically significant. To assess the level of knowledge on malaria prevention strategies, respondents were scored 1 if they had knowledge of a strategy and were scored of 0 if they had no knowledge of it. A total score of 80% and above was graded as good knowledge, 60%-79% as satisfactory knowledge, and a grade below 60% as poor knowledge.

### 2.6. Ethical Considerations

Ethical approval for the study was obtained from the University of Health and Allied Sciences Research Ethics Committee (UHAS-REC/A.3 (17) 17-18). Permission was also sought from the elders of the Godokpe community. All procedures required for the protection of human participants were adopted. Respondents who agree to participate in this study were made to sign or thumbprint a written informed consent before the commencement of data collection. Respondents' names were not required in completing the research tool. Data was precoded such that each response could not be traced to a specific respondent. Data was entered into a secured computer where only persons on the research team had access to.

## 3. Results

Females constituted the majority of respondents recruited for the study (52.0%). Respondents aged 26 to 40 years formed the majority (42.3%) while those 65+ years were the least age group represented (6.4%). Most of the respondents had been to school before; however, majority (44.4%) ended their education at the primary level (Table 1).

### 3.1. Awareness on Malaria Spread, Symptoms, and Treatment

As shown in Table 2, it was revealed that 98% of the respondents were aware of malaria but 40% thought malaria was transmissible from an infected person to a noninfected person. Common symptoms of malaria were identified by majority of the respondents (88% and 87%) as shivering and fever, respectively. Nausea, on the other hand, was indicated by only 51% of the respondents as a common symptom. More than two-thirds of the respondents (75%) identified correctly female anopheles' mosquito as the malaria vector. About 70% of the respondents indicated that quinine was used for malaria treatment, while 50% also identified chloroquine as the medicine used to treat malaria. Amongst the 22 respondents who indicated that there were other medicines for malaria treatment, almost all of them (96%) indicated ACT as the treatment for malaria. More than half of the respondents (53%) indicated that malaria was a deadly disease while 39% thought malaria was an ordinary disease.

### 3.2. Knowledge on Preventive Measures

The respondents generally indicated that standing clean water (34%) and garbage/trash (33%) are the commonest breeding sites. The time for most frequent mosquito bite was identified by most respondents (71%) as the night and only 4% thought that most frequent mosquito bites were in the morning. As shown in Table 3 majority of the respondents had knowledge of different preventive measures such as use of mosquito net (93%), mosquito mat, coils and liquid vaporizer (90%), and mosquito spray (84%). The preventive measures which less than half of the respondents had knowledge on were the use of fan (43%) and covering of the body with clothes (36%). It was revealed that most of the respondents (84%) had knowledge on the use of prevention of water stagnation as a strategy to eradicate the breeding sites of mosquitoes as indicated in Table 3.

### 3.3. Strategies Used in Malaria Prevention

In Table 4, it was found that the main strategy for malaria prevention was the use of mosquito mats, coils, and liquid vaporizers (72%). More than half of the respondents also used water stagnation prevention (62%), cleaning house (62%), mosquito net (60%), and mosquito spray (54%) as their strategies for malaria prevention. The least recorded strategy, however, was the use of smoke to drive away mosquitoes (32%).

Respondents made varied responses regarding knowledge and use of ITN as a preventive measure. There were great differences in knowledge and use amongst measures such as mosquito spray, mosquito net, and water stagnation prevention. For mosquito spray, 83% of the respondents had knowledge but only 54% used it. Similarly, 93% had knowledge on mosquito net usage but only 59% used it for malaria prevention. Prevention of water stagnation is known by 83% of the respondents; however, only 62% used it as a strategy for malaria prevention. It was revealed although 36.0% of the respondents had knowledge on covering of the body with clothes as a strategy for malaria prevention, about 50.0% of the respondents used it for malaria prevention.

The findings revealed that television was the commonest source amongst most of the respondents (74%). More than half of the respondents also had their source of information from health professionals (65%), schools (62%), family/friends (60%), and the Internet (51%). The newspaper was the source of information assessed by only a few of the respondents (19%).

### 3.4. Association between Knowledge of Malaria Preventive Measure and Demographic Characteristics

Table 5 showed the association between knowledge of malaria preventive measures and demographic characteristics. About 22% of the respondents between ages 41 and 64 years had good knowledge on malaria preventive measures followed by 21% of those 18 to 25 years, 20% of those 26 to 40 years, and 6% of those 65+ years who had good knowledge. More females (20%) had good knowledge on malaria preventive measure than males (19%); however, the association was not statistically significant. Most of the people who had good knowledge on malaria preventive measure were the uneducated (24%). None of those with tertiary education had good knowledge on malaria preventive measures.

## 4. Discussion

This study assessed the knowledge of the people of Godokpe in the Ho municipality on the presentation of malaria and control measures and the methods adopted by each individual in preventing malaria. Majority (98%) of the respondents were aware of malaria as 40% indicated malaria was transmissible from an infected person to a noninfected person. In line with this, the National Malaria Control Programme, University of Health and Allied Sciences, AngloGold Ashanti Malaria Control Program, and others, 2013 [12], argued that the population's knowledge about malaria and its etiology has drastically changed and that communities today are aware that mosquitoes transmit malaria and malaria is a serious febrile disease. Majority of the respondents (75%) identified female anopheles' mosquito as the malaria vector. This knowledge remains central to the methods respondents adopt in preventing contact with the vector especially at pike transmission times. Similarly, Kwaku [13] found that 89.1% of respondents were aware that malaria was transmitted through mosquito bite. Also Adongo et al. [14] found that 79% of their study respondents indicated mosquitoes as the cause of malaria. Common symptoms of malaria included shivering (88%) and fever (87%). Nausea, on the other hand, was indicated by 51% of the respondents as a common symptom. In Northern Ghana, Adongo et al. [14] showed that common symptoms they reported on were hot body (97%), vomiting (38%), chills (27%), headache (26%), and loss of appetite (8%).

With regard to malaria treatment amongst the residents of Godokpe, we found that about 70% of the respondents used quinine and 50% also used chloroquine. The Ghana health service recommends the use of artemisinin based combination therapy for the treatment of uncomplicated malaria and quinine for the treatment of complicated malaria. Buabeng, 2010 [15], found that 76% of the study participants who self-medicated for malaria therapy used chloroquine. Another treatment used by the residents is the use of Artemisinin-based combination therapy (ACT). The use of this artemisinin-based combination therapy is in line with the policy of the Ghana health service using this as first line treatment for uncomplicated malaria.

Malaria still remains a disease of public health importance in Ghana and among the leading causes of death in Sub-Saharan Africa, although it is curable and preventable [16]. While knowledge on bed net as a preventive measure was high (93%), the actual usage of the net was identified to be low (60%). Community members do not actually translate knowledge to actual practice. Elsewhere, Azabre et al. [17] found that 62.1% of the study participants had adequate knowledge on ITNs. Similarly, Adjei, 2012 [18], found 48% of the households owning bed net. On the other hand, ownership of insecticide treated net is reported as 68% in households in Ghana (Ghana Statistical Service [GSS], Ghana Health Service [GHS], & ICF International 2015); however, current malaria control strategy targets 100% of household ITN ownership. The use of ITN is generally seen as one of the most effective strategies in malaria prevention; hence, in Ghana, there is a regular distribution of ITNs amongst the population and more especially pregnant women are freely given the ITN when they visit the health facilities.

The use of mosquito net, coils, and liquid vaporizer (72%) was the most used strategy in malaria prevention. Binka and Smith [19] argued that ITNs reduce malaria prevention by 90% and as well reduce indoor vector population. Kwaku, 2015, found that most of the respondents also used mosquito coil as malaria prevention strategy. Prevention of water stagnation and cleaning of the house were both used in malaria control (62%). Mosquito spray was also known by 84% but only 54% of the respondents used it in malaria prevention. Azabre et al., 2013, found that 47.1% of their study participants had knowledge on environmental sanitation/clearing of bushes as methods of malaria prevention. Azabre et al. [17] reported that 44.3% of individuals use wearing of long clothes/boots to prevent mosquito bites, which is consistent with this study's findings that 50% of the study participants used clothing as a strategy to prevent mosquito bites leading to malaria.

Usage of mosquito sprays as malaria control strategy was reported by 54%. The 2014 Ghana Demographic and Health Survey reported that 10% of households had indoor residual spraying (IRS) done within the 12 months preceding the survey. The National Malaria Control Program was with the objective of providing IRS to at least 90% of all houses in targeted districts by 2011 and to sustain this coverage through to 2015 (National Malaria Control Programme, University of Health and Allied Sciences, AngloGold Ashanti Malaria Control Programme, and others, 2013). Usage of fan to drive mosquitoes away was also reported by 38%. Azabre et al. [17] found only 12.1% who used fans to drive mosquitoes and 8% who used mosquito coils and sprays. The least used strategy for malaria prevention revealed in this study was the use of smoke to drive away mosquitoes (32%). Azabre et al., 2013, also found 33.3% of their study participants who used burning of weeds as traditional repellent.

The commonest source of information on malaria prevention was television (74%). The Internet also served as a source of information for nearly half of the respondents (51%). Bello-Bravo et al. [20] found that most of their study participants (75%) used the Internet as means for finding information about malaria and majority also indicated that their source of information and resources about malaria were through clinics, hospitals, and doctors; in turn, this corresponds with this study findings that revealed that more than half of the respondents also had their source of information from health professionals (66%). Kwaku [13] found health workers (79%) as the major source of information on malaria transmission and ITN usage. Television was the source of information for only 6% while relatives/friends formed 4%. Other sources of information on malaria prevention identified by this study were schools (62%), family/friends (60%), and newspaper (19%).

This result basically portrays a cross-sectional view of the people of Godokpe community regarding the spread of malaria and measures adopted in its prevention. Several interventions have been instituted in the Godokpe community by public health nurses and community health nurses with the sole prerogative of preventing the continuous spread of the malaria parasite. A peri-urban community generally in Ghana is also more concerned about health and may take in measure towards attaining the same. Also, the use of systematic sampling method was relatively problematic as in some instances respondents were not readily available at home to respond to the questionnaire.

## 5. Conclusion

The residents of Godokpe had satisfactory knowledge on malaria and malaria prevention strategies. Common symptoms of malaria were revealed by most residents as shivering, fever, vomiting, headache, and body pains. Also, malaria treatments used by the residents of Godokpe were quinine, chloroquine, and ACT. The preventive strategies used by most residents in Godokpe were the use of mosquito mats, coils, and liquid vaporizer, prevention of stagnant water, cleaning of houses, use of mosquito nets, and the usage of spray. The most common sources of information on malaria and malaria prevention strategies were television, health professional, schools, friends/families, and the Internet. This study basically elicited the level of knowledge of a peri-urban rural community in Ghana on malaria. The hope is that this information will be useful in tailoring health education and interventions strategies in preventing the malaria menace in Ghana. There is the need to educate the people of Godokpe on the essence to seek early healthcare services when they identify any signs and symptoms of malaria. School children have shown good understanding of malaria and its vectors; therefore, there is a need to empower teachers with appropriate health information including malaria so that they can deliver malaria information to the pupils. The Ho Municipal Health Directorate should educate the people of Godokpe on the importance of ITN usage and indoor residual spraying to ensue effective prevention of malaria.

Residual malaria transmission is a new challenge which is sufficiently intense across most of the tropical countries which render malaria elimination to be unfeasible; thus information, educational, and communication (ICE) facilities, such as television, radio, posters, schools, and health centres, could be used to disseminate information of changed feeding behaviour of malaria vector so that people can protect themselves against early outdoor mosquito bite by using mosquito repellents, long sleeved cloths, and environmental manipulations.

## Figures and Tables

**Table 1:** Sociodemographic characteristics of respondents.

Variables	Attributes	Frequency (n=246)	Percent (%)
Age of respondents	18-25 years	76	31
	26-40 years	105	42
	41-64 years	51	21
	65+ years	16	6

Sex of respondents	Male	119	48
	Female	129	52

Highest Educational level ever attained by respondents	None	54	22
	Primary	110	44
	Secondary	73	29
	Tertiary	11	4

**Table 2:** Awareness on malaria spread, symptoms, and treatment.

Variable	Attributes	Frequency (n=246)	Percent (%)
Awareness of malaria	Yes	242	98
	No	6	2

Malaria is transmissible from infected person	Yes	100	40
	No	148	60

Common symptoms of malaria	Fever	215	87
	Nausea	126	51
	Headache	179	72
	Body aches	167	67
	Vomiting	181	73
	Shivering	219	88

Name of vector that transmits malaria	Female anopheles	187	75
	Male anopheles	7	3
	No idea	54	22

Medicines against malaria	Chloroquine	125	50
	Quine	173	70
	No idea	2	1
	Others	22	9

Other forms of malaria treatment	ACT	21	96
	Artemether lumefantrine	1	5

Malaria is a/an	Ordinary disease	96	39
	Deadly disease	132	53
	No idea	20	8

**Table 3:** Knowledge of vector characteristics and preventive measures.

Variable	Attributes	Frequency (n=246)	Percent (%)
Common breeding site	Running dirty water	36	15
	Garbage/trash	81	33
	Standing clean water	84	34
	Standing dirty water	38	15
	Running clean water	3	1
	Plants/Vegetation	6	2

Most frequent mosquito bite time	Sunset/Dusk	47	19
	Sunrise/Dawn	15	6
	Morning	10	4
	Night	176	71

Knowledge of preventive measures	Use of smoke to drive away mosquitoes	64	26
	Mosquito mat, coils, and liquid vaporizer	222	90
	Mosquito spray	208	84
	Use of fan	107	43
	Covering of body with clothes	89	36
	Mosquito net	231	93
	Cleaning house	138	56
	No idea	2	1

Eradication of breeding site of mosquito	Prevent water stagnation	208	84
	Covering containers	112	45
	Changing water in storage tanks	142	57
	Others	17	7
	No idea	4	2
	Proper disposal of waste	17	7

**Table 4:** Strategies/materials used in malaria prevention.

Variables	Frequency (N=246)	Percent (%)
Use of smoke to drive away mosquitoes	80	32
Mosquito mat, coils, and liquid vaporizer	178	72
Mosquito spray	134	54
Use of fan	93	38
Covering of body with clothes	124	50
Mosquito net	148	60
Cleaning house	154	62
Prevent water stagnation	154	62
Covering containers	115	46
Changing water in storage tanks	123	50
Others	10	4
No idea	4	2

**Table 5:** Association between knowledge of malaria preventive measure and demographic characteristics.

		Knowledge on malaria preventive measures			
Variable	Responses	Poor knowledge (%)	Fair knowledge (%)	Good knowledge (%)	*P* value
Age of respondents	18-25 years	20(26)	40(53)	16(21)	0.110
	26-40 years	20(19)	64(61)	21(20)	
	41-64 years	20(39)	20(39)	11(22)	
	65+ years	5(31)	10(63)	1(6)	

Sex	Male	38(32)	58(49)	23(19)	0.131
	Female	27(21)	76(59)	26(20)	

Highest educational level attained by respondents	None	14(26)	27(50)	13(24)	0.094
	Primary	24(22)	65(59)	21(19)	
	Secondary	20(27)	38(52)	15(201)	
	Tertiary	7(64)	4(36)	0(0)	

## Data Availability

All discussions and conclusions in this paper are based on primary data. All data based on which the conclusion of this paper is based are all stated in this paper; there are no data deposited in any repository.

## References

[1] Bhatt S., Weiss D. J., Cameron E. (2015). The effect of malaria control on Plasmodium falciparum in Africa between 2000 and 2015. *Nature*.

[2] Weil D. N. (2015). A review of angus deaton's the great escape: health, wealth, and the origins of inequality. *Journal of Economic Literature (JEL)*.

[3] González R., Sevene E., Jagoe G., Slutsker L., Menéndez C. (2016). A public health paradox: the women most vulnerable to malaria are the least protected. *PLoS Medicine*.

[4] National Malaria Control Programme-NMCP (2014). *National Malaria Control Programme Report*.

[5] Kimbi H. K., Nkesa S. B., Ndamukong-Nyanga J. L., Sumbele I. U. N., Atashili J., Atanga M. B. S. (2014). Knowledge and perceptions towards malaria prevention among vulnerable groups in the Buea Health District, Cameroon. *BMC Public Health*.

[6] Heggenhougen H. K., Hackethal V., Vivek P. The behavioural and social aspects of malaria control: an introduction and annotated bibliography.

[7] Adera T. D. (2003). Beliefs and traditional treatment of malaria in Kishe settlement area, South West Ethiopia. *Medical Journal*.

[8] Nkuo Akenji T. K., Ntonifor N. N., Ching J. K. (2005). Evaluating a malaria intervention strategy using knowledge, practices and coverage surveys in rural Bolifamba, southwest Cameroon. *Transactions of the Royal Society of Tropical Medicine and Hygiene*.

[9] Abubakari A., Karim A., Saeed B. I. I., Afrifa-Yamoah E. (2016). Trend analysis of malaria cases in Ghana from 1985-2008. *Sky Journal of Medicine and Medical Sciences*.

[10] Ghana Health Service (GHS) (2017). *National Malaria Control Programme*.

[11] Awine T., Malm K., Bart-Plange C., Silal S. P. (2017). Towards malaria control and elimination in Ghana: challenges and decision-making tools to guide planning. *Global Health Action*.

[12] National Malaria Control Program (2013). *An Epidemiological Profile of Malaria and Its Control in Ghana*.

[13] Kwaku B. M. (2015). *Ownership and Utilization of Insecticide Treated Nets (ITNS) among Pregnant Women and Children under Five Years in the Prevention of Malaria in the Nanumba South District of The Northern Region of Ghana*.

[14] Adongo P. B., Kirkwood B., Kendall C. (2005). How local community knowledge about malaria affects insecticide-treated net use in northern Ghana. *Tropical Medicine & International Health*.

[15] Buabeng K. O. (2010). *The Role of the Pharmaceutical Sector*.

[16] Ghana Demographic and Health Survey (2014). *Demographic and Health Survey*.

[17] Azabre B. A., Teye J. K., Awetoriyaro J. (2013). Malaria control strategies in the Kassena-Nankana East and West Districts of Ghana. *Ghana Journal of Geography*.

[18] Adjei J. K., Gyimah S. O. (2012). Household bednet ownership and use in Ghana: implications for malaria control. *Canadian Studies in Population*.

[19] Binka F., Smith T. A. (1998). Impact of spatial distribution of permethrin- impregnated bed nets on child mortality in rural northern Ghana. *The American Journal of Tropical Medicine and Hygiene*.

[20] Bello-Bravo J., Lutomia A. N., Madela L. M., Pittendrigh B. R. (2017). Malaria prevention and treatment using educational animations: a case study in Kakamega County, Kenya. *International Journal of Education and Development Using Information and Communication Technology*.

